# Excessive EFHD1-dependent ER-mitochondrial contacts drive a maladaptive antiviral response in metabolic liver disease

**DOI:** 10.1172/JCI204023

**Published:** 2026-09-01

**Authors:** David R. Eberhardt, Emma C. Rekate, Yasmin B. Masini, Hannah E. Duron, David Mollinedo, Adrian M. Velarde, Devorah Stucki, Tara R. Price, Sandra H.J. Lee, Enrique Balderas, Neeraj K. Rai, Ashley R. Bratt, Anthony M. Balynas, Chris J. Stubben, Ryan Bia, Sudipa Maity, Nicolas Hartel, Xue Yin, Andrea Corbin, Anshu Kumari, Dung M. Nguyen, Daisuke Shimura, Vu D. Nguyen, Vishaka Vinod, Kamrul H. Chowdhury, Francisco Verdeguer, Joel Zvick, Patrice N. Mimche, Sihem Boudina, Stavros G. Drakos, Ademuyiwa S. Aromolaran, Sarah Franklin, Vivek Garg, Robin M. Shaw, William L. Holland, Scott A. Summers, Marcus G. Pezzolesi, Jared Rutter, Kimberley J. Evason, Dipayan Chaudhuri

**Affiliations:** 1 Nora Eccles Harrison Cardiovascular Research and Training Institute and; 2 Department of Internal Medicine, Division of Nephrology and Hypertension, University of Utah, Salt Lake City, Utah, USA.; 3 Bioinformatics Shared Resource, Huntsman Cancer Institute (HCI), Salt Lake City, Utah, USA.; 4 Thermo Fisher Scientific, San Jose, California, USA.; 5 Department of Pharmacology and Physiology, University of Maryland School of Medicine, Baltimore, Maryland, USA.; 6 Department of Nutrition and Integrative Physiology, University of Utah, Salt Lake City, Utah, USA.; 7 InSphero AG, Schlieren, Switzerland.; 8 Department of Dermatology, Indiana University School of Medicine, Indianapolis, Indiana, USA.; 9 Department of Internal Medicine, Division of Cardiovascular Medicine;; 10 Department of Surgery, Division of Cardiothoracic Surgery;; 11 Departments of Biochemistry and Biomedical Engineering;; 12 Department of Human Genetics;; 13 Howard Hughes Medical Institute, Department of Biochemistry; and; 14 Department of Pathology and HCI, University of Utah, Salt Lake City, Utah, USA.

**Keywords:** Cell biology, Hepatology, Metabolism, Calcium signaling, Cellular immune response, Mitochondria

## Abstract

Metabolic dysfunction–associated steatohepatitis (MASH) involves hepatocyte damage that cannot be explained solely by lipid accumulation. Here, to discover injury-specific pathways, we focused on a gene of uncertain function, EF-hand domain family member D1 (EFHD1), identified in human genome-wide association studies of liver injury but not liver fat. We showed that EFHD1, a Ca^2+^-dependent actin cross-linker, stabilizes endoplasmic reticulum–mitochondria contact sites (ERMCS) by detecting spatiotemporal coincidence of interorganellar proximity and ER Ca^2+^ release. During MASH, EFHD1 upregulation drives pathological mitochondrial fragmentation via excessive contact persistence. This structural failure promotes mitochondrial double-stranded RNA escape and activation of a maladaptive, antiviral, protein kinase RNA-activated–associated stress response, a causal relationship also supported by Mendelian randomization in humans. Consequently, inhibiting EFHD1 in human and mouse models blunts hepatocyte damage. These findings identify EFHD1 as a Ca^2+^-dependent ERMCS stabilizer, reveal a hepatocyte-intrinsic injury pathway, and suggest EFHD1 inhibition as a therapeutic strategy.

## Abstract 2

In MASH, a liver disease linked to obesity, the protein EFHD1 makes cells mistakenly react as if infected by a virus. Blocking EFHD1 prevents damage.

## Graphical abstract

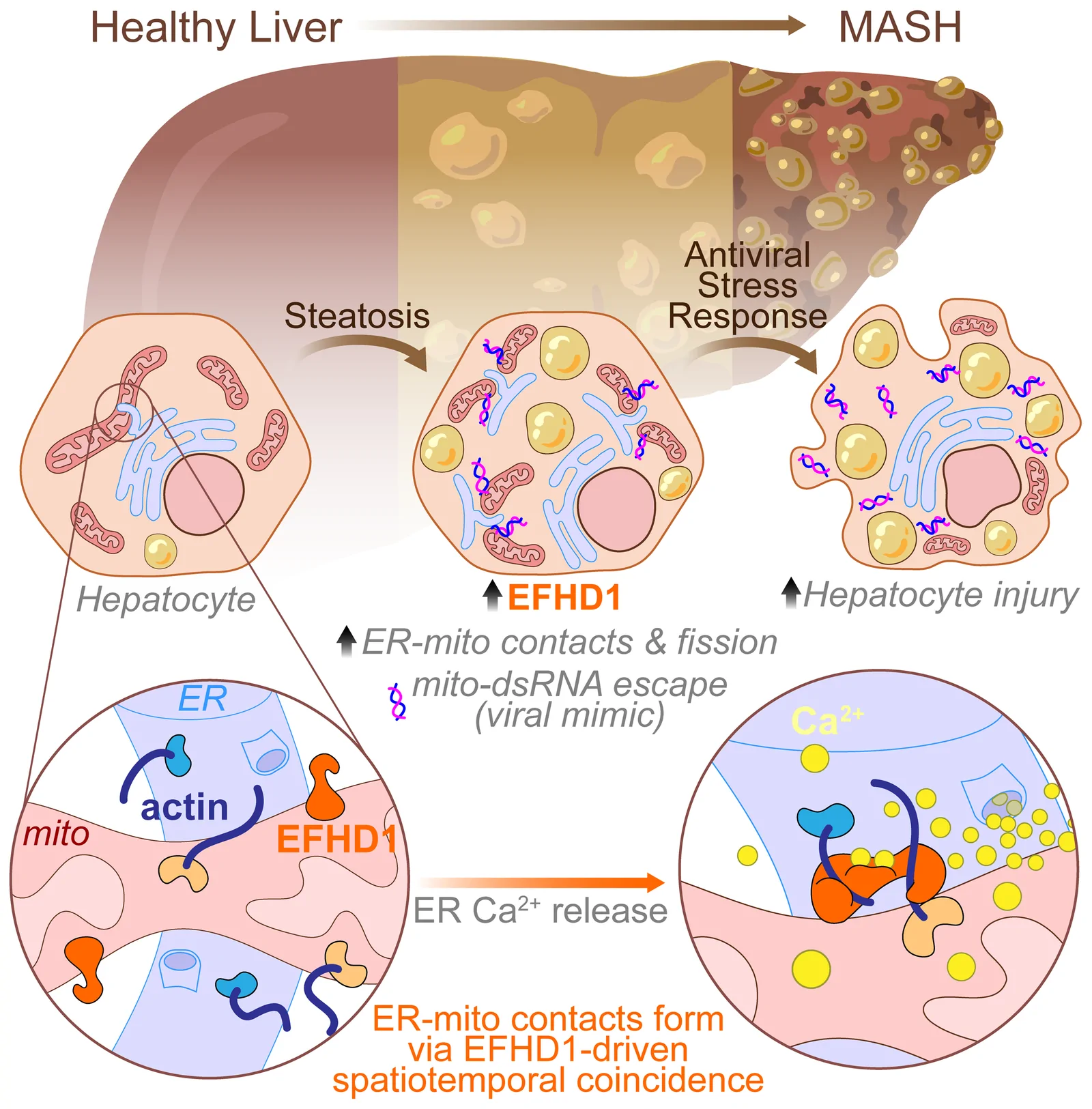

## Introduction

The growing prevalence of metabolic dysfunction–associated steatotic liver disease and steatohepatitis (referred to hereafter as MASH) is an area of profound unmet clinical need (1). One approach for therapeutic and mechanistic insight has been to seek genes altering MASH risk (2, 3). Multiple genome-wide association studies (GWAS) for MASH have assessed either liver fat content or susceptibility to liver injury. Both types of analyses have repeatedly identified genes critical for hepatic lipid metabolism. However, lipid accumulation alone does not predict progression to MASH, and therapies inhibiting lipid metabolism have been hampered by difficulties (4–6). Therefore, to identify pathways that contribute to liver injury independently of lipid metabolism, we focus here on a gene of unknown function repeatedly identified in GWAS of liver enzymes, but not in GWAS measuring liver fat, EF-hand domain family member D1 (EFHD1) (7–10).

EFHD1 is a 27 kDa, nuclear-encoded protein that localizes to mitochondria and is expressed widely. Variation in the *EFHD1* locus has been consistently associated with serum levels of liver enzymes, markers of liver injury, across multiple populations (7–10). The variant most strongly associated with elevated serum liver enzymes increases liver-specific expression of *EFHD1* by encoding a better consensus sequence for hepatic transcription factors, including FOXA2, FOXO1, and HNF4A (11–13). However, modulating EFHD1 does not produce a consistent effect on glycolysis or fatty acid metabolism across different cell types (12–15). Thus, whether EFHD1 truly contributes to MASH progression, and by what mechanism, remain critical unanswered questions.

Here, using mouse and human models, we show that EFHD1 contributes to a central pathway for hepatocyte injury. Notably, this occurs without altering organismal energy balance or hepatic steatosis, consistent with human GWAS linking *EFHD1* to liver enzymes but not fat, and suggesting that the EFHD1-dependent injury is independent of the initial insult. Mechanistically, we identify that EFHD1, a Ca²^+^-dependent actin cross-linker, functions as a spatiotemporal coincidence detector, converting transient ER Ca²^+^ signals into mechanically persistent ER–mitochondria coupling. During metabolic stress, EFHD1 upregulation leads to pathological contact persistence and aberrant mitochondrial fission. These events promote cytoplasmic release of mitochondrial double-stranded RNA (mt-dsRNA) and activation of a maladaptive integrated stress response, a pathway normally reserved for antiviral defense. Finally, EFHD1 inhibition blunted hepatocyte injury, inflammation, and fibrosis across multiple metabolic and chemical liver disease models, suggesting it is a tractable target for selectively uncoupling liver injury from lipid accumulation.

## Results

### EFHD1 is expressed in hepatocytes and increases during MASH.

In humans, EFHD1 is preferentially expressed in hepatocytes within the liver, with enrichment in periportal hepatocyte clusters with high complement and immune pathway activation (Figure 1, A and B, and Supplemental Figure 1A; supplemental material available online with this article; https://doi.org/10.1172/JCI204023DS1) (13, 16–20). Moreover, we found increased liver EFHD1 in humans with MASH (Figure 1, C and D). To investigate EFHD1 function further, we treated mice with 3 separate diets, a normal chow diet (10% fat); a high-fat diet (HFD, 60% fat, 18% protein, 22% carbohydrate), which produces obesity but only mild liver injury; and a Gubra Amylin MASH diet (40% fat, 2% cholesterol, 20% fructose), which mimics hallmarks of human MASH injury, including hepatocyte ballooning, inflammation, and fibrosis (21, 22). For HFD and MASH, mice were started on the diet at 8 weeks of age for up to 28 weeks’ duration (36 weeks of age) (Supplemental Figure 1B). EFHD1 increased 2- to 4-fold in wild-type (WT) animals on both HFD and MASH diets (Figure 1, E and F, and Supplemental Figure 1, C and D). Taken together, human and mouse studies suggest EFHD1 is expressed in hepatocytes and is upregulated during overnutrition.

### Deletion of EFHD1 is protective in MASH without affecting energy balance.

Because GWAS implicate EFHD1 in liver injury without specifying the causal tissue, we examined whole-body EFHD1-knockout mice (*Efhd1^–/–^*) (Figure 1, G and H) (23). The mice exhibited normal weight gain, body composition, feeding, activity, insulin resistance, and liver/body weight ratio (Supplemental Figure 1, E–R), establishing that loss of EFHD1 is well tolerated and that changes in hepatic physiology are independent of global energy balance.

To examine EFHD1 effects on MASH progression, we measured a range of serum and histological biomarkers of hepatic function in mice after a 4-hour fast. First, we assayed hepatic lipid metabolism. In HFD and MASH, *Efhd1^–/–^* males exhibited lower levels of serum triglycerides (Figure 1I and Supplemental Figure 2, A and B). Next, a pathologist without knowledge of genotype assessed the overall histological MASH activity score, as well as the individual hepatocyte ballooning/hypertrophy, steatosis, and inflammation scores (Figure 1, J–M, and Supplemental Figure 2, C–G and J–L) (24, 25). We complemented this with direct measurement of lipid droplet size and count (Figure 1, N and O, and Supplemental Figure 2, H, I, and M–Q). Interestingly, though there were differences between genotypes, primarily for males, in ballooning and lipid content during chow diet, these differences disappeared under the obesogenic diets. The diet-dependent variability in these results suggests EFHD1 affects hepatic lipid metabolism indirectly and is consistent with the absence of *EFHD1* in human MASH GWAS studies that measure hepatic fat.

In contrast, injury markers were markedly reduced in *Efhd1^–/–^* across diets, assessed both via serum liver enzymes and via histology (Figure 1, P–W, and Supplemental Figure 2, R–Z). Serum AST and ALT levels in *Efhd1^–/–^* mice were about half of WT (Figure 1, P and Q, and Supplemental Figure 2, R–U). Liver inflammation and fibrosis were also reduced in males on both histopathological scoring and direct measurement (Figure 1, R–W, and Supplemental Figure 2, V–Z). In *Efhd1^–/–^* livers, we also noted fewer inflammatory cells per cluster (Figure 1R and Supplemental Figure 2V). Female mice are less injured by overnutrition (26), so they had minimal inflammation, though inflammatory clusters and fibrotic area were still reduced in MASH *Efhd1^–/–^* females (Figure 1, T and W). Fibrosis was minimal for chow and HFD and not scored (Supplemental Figure 2W). To summarize, loss of EFHD1 is protective during MASH progression due to reduced liver injury.

### EFHD1 is necessary for Ca^2+^-induced mitochondrial remodeling.

Because EFHD1 localizes to the outer mitochondrial membrane (OMM), including ER-mitochondrial contact sites (ERMCS) (Supplemental Figure 3, A and B), we tested its contribution to mitochondrial morphology (23, 27–30). We examined mouse hepatocytes within 24 hours of isolation, prior to dedifferentiation (Supplemental Figure 3, C–E), using MitoTracker Orange. Intriguingly, whereas WT mitochondria appeared bean shaped, *Efhd1^–/–^* mitochondria were longer and spaghetti shaped (Figure 2, A and B). MitoTracker Orange only labels polarized mitochondria, confirming this was not due to differences between injured versus healthy mitochondria. This difference was notable even in hepatocytes isolated from mice subjected to MASH diet, which causes extreme mitochondrial fission (Figure 2C). Moreover, the difference in mitochondrial length was also evident on transmission electron micrography (TEM) and via staining for mitochondria in fixed cells (Figure 2, D–F, and Supplemental Figure 3, F and G), confirming the effect was not due to differential staining by the MitoTracker dye. Finally, this effect was not hepatocyte specific, could be replicated in cultured *EFHD1^–/–^* HepG2 and HAP-1 cells, and could be rescued by reexpression of EFHD1 (Figure 2, G–I, and Supplemental Figure 3, H–K) (31).

That mitochondrial remodeling is associated with ER Ca^2+^ release was established more than 20 years ago, but its significance, mechanism, and sensor remain elusive (32, 33). Therefore, we tested whether EFHD1 mediates Ca^2+^-dependent mitochondrial remodeling, by triggering inositol triphosphate receptor–mediated ER Ca^2+^ release with 1 mM extracellular ATP (Figure 2, A, B, G, and H) (34). This led to a marked reduction in size in WT but not *Efhd1^–/–^* mitochondria. Next, we focused on mitochondrial fission, a well-defined outcome of stable ERMCS formation (35). We found that deleting EFHD1 in both hepatocytes and HepG2 cells prevented the increase in fission events following ATP treatment (Figure 2, J and K). Moreover, fission was also reduced at baseline in EFHD1-deficient cells. To confirm Ca^2+^ dependence, we permeabilized plasma membranes with a low concentration of digitonin, leaving mitochondria intact, and added a 100 μM Ca^2+^ bolus. In WT cells, mitochondria both rounded up and fissioned, an effect substantially blunted in *Efhd1^–/–^* cells (Figure 2, L and M). This blunting of fission could also be replicated with direct application of the Ca^2+^ ionophore ionomycin (Supplemental Figure 3L). Finally, this effect was linked to nutritional state. Adding glucose and palmitate to starved hepatocytes remodeled WT but not *Efhd1^–/–^* mitochondria (Supplemental Figure 3M). Our results add to the growing body of evidence connecting ER-mitochondrial ion transfer to metabolism (36–38). Taken together, these results establish EFHD1 as a necessary transducer of ER Ca^2+^ release into a mitochondrial remodeling signal.

### EFHD1-dependent actin bundling integrates spatial proximity with a temporal Ca^2+^ signal to stabilize ERMCS.

Next, we investigated how EFHD1 drives Ca^2+^-dependent remodeling. When ERMCS form, actin filaments are thought to assemble by an interaction between the ER-associated protein INF2 and mitochondria-associated Spire1C (Figure 3A) (39, 40). For subsequent fission, the cytoplasmic GTPase DRP1 is recruited by adaptors and constricts the mitochondrion until it divides. Prior studies have suggested Ca^2+^ can drive INF2-dependent actin polymerization, aid in the recruitment of DRP1 to fission sites during cell injury, and drive energetics within the mitochondrial matrix (41–44). Thus, how this Ca^2+^ signal drives remodeling remains undefined.

We began by testing whether the Ca^2+^ signal for remodeling acts at the OMM or within mitochondria (41). In all the experiments described so far, we had incubated cells with 1 μM Ru360, a blocker of the mitochondrial Ca^2+^ uniporter, the channel conducting Ca^2+^ into the matrix, implying that remodeling did not require Ca^2+^ uptake. To confirm this further, we silenced the main uniporter subunit, MCU, with short hairpin RNA (Supplemental Figure 4A) (45) in *EFHD1^–/–^* or EFHD1-overexpressing cells, allowing us to test whether MCU affects fission differentially at the extremes of EFHD1 expression. In fact, we found that depleting MCU had no effect on ATP-induced fission events (Supplemental Figure 4B). Because prior work suggested EFHD1 can bind the mitochondrial Ca^2+^ uniporter, we also examined whether EFHD1 modulates hepatocyte mitochondrial Ca²^+^ entry (30). Across assays of uniporter protein levels, mitochondrial Ca^2+^ uptake, and mitoplast patch-clamp measurements, we detected no differences between WT and *Efhd1^–/–^* hepatocytes (Supplemental Figure 4, C–M). The imaging assays with ATP (Supplemental Figure 4, G–I) also confirmed that differences in remodeling seen in Figure 2 were not due to aberrant ER Ca^2+^ release. Thus, the Ca^2+^ signal driving EFHD1-dependent mitochondrial remodeling localizes to the OMM.

Next, we tested the hepatic expression levels of proteins involved in mitochondrial dynamics (Figure 3B and Supplemental Figure 5A). The most prominent change was a marked increase in DRP1 in *Efhd1*^–/–^. This was not due to changes in mitochondrial content, as mitochondrial markers NDUFS3 and TOMM20 were unchanged. This result is counterintuitive, since increased DRP1 would be expected to produce excessive fission in *Efhd1^–/–^*, rather than the elongated mitochondria we observe. An explanation might be that DRP1 is not migrating to the OMM, since prior studies established that, during cell damage, Ca^2+^ promotes DRP1 localization to the OMM by enhancing Ser616 phosphorylation and Ser627 dephosphorylation (43, 44). Consequently, we examined DRP1 OMM recruitment. Unexpectedly, DRP1 Ser616 phosphorylation was increased, while Ser637 phosphorylation was reduced in *Efhd1^–/–^* livers, suggesting DRP1 was hyperactive (Figure 3B). Moreover, DRP1 localized to mitochondria in both WT and *Efhd1^–/–^* cells by imaging and Western blot, further confirming no defect in its activity (Supplemental Figure 5, B and C). Therefore, EFHD1 is not directly affecting mitochondrial fission. Rather, the increased expression of hyperactive DRP1 likely contributes to the residual fission still present in *Efhd1^–/–^* cells, and along with the decreased expression of fusion proteins MFN2 and OPA1 (Figure 3B), forms a compensatory response for an impairment at an earlier step in ERMCS formation.

Since EFHD1 is not directly involved in DRP1 activation or downstream fission, we examined Ca^2+^-dependent actin rearrangements at ERMCS (39). In fact, EFHD1 and its homolog EFHD2 are known to bundle actin filaments in the presence of Ca^2+^ (46, 47). Moreover, in recent protein-protein interaction compendia, EFHD1 is annotated as an actin-binding protein, which we confirmed via co-immunoprecipitation (Figure 3C and Supplemental Figure 5D) (29). Expression of INF2, which polymerizes actin at ERMCS but does not itself bind Ca^2+^, was also reduced in *Efhd1^–/–^*, though it did not co-immunoprecipitate with EFHD1 (Figure 3, B and C). Next, we modeled binding via AlphaFold3, which predicted a high-confidence interaction between EFHD1 and β-actin (Figure 3D and Supplemental Figure 5E) (48). In the highest confidence hypothetical structure, EFHD1 was primarily bound between domains 1 and 2 of actin, leaving free the plus and minus ends, similar to other actin-bundling proteins (Supplemental Figure 5F) (49, 50). The greatest uncertainty in the prediction was the localization of the EFHD1 initial helical domain, with several of the top 5 hypothetical models suggesting it could interact with the actin plus end, potentially regulating actin polymerization (Supplemental Figure 5E).

We then investigated whether loss of EFHD1 would alter actin networks, ERMCS stability, and Ca^2+^-dependent actin remodeling. First, whereas control cells had diffuse cytoplasmic actin staining, cells lacking EFHD1 displayed prominent cytoplasmic actin stress fibers, away from peripheral cortical networks (Figure 3E and Supplemental Figure 5, G and H). Second, we examined ERMCS, where mitochondria and ER are less than 30 nm apart (Figure 3, F and G) (51). We analyzed electron micrographs in 2 ways, measuring either the distance at ERMCS below the 30 nm threshold, or distances whenever there were stretches of ER and OMM < 500 nm apart. Notably, in animals fed a chow diet, ERMCS were narrower for *Efhd1*^–/–^ hepatocytes compared with WT, suggesting ER and mitochondria needed to be closer to establish robust contacts. In addition, while ERMCS distances were stable between WT animals fed either a normal or MASH diet, ER-mitochondrial distances were widened and irregular in *Efhd1^–/–^* hepatocytes in MASH, including distinct populations with large separations not present at baseline (Figure 3G). The failure to maintain ERMCS integrity during MASH indicated that EFHD1 is required for stabilizing these contacts (52). Next, to directly test EFHD1 contribution to ERMCS stability, we employed live-cell imaging using the splitFAST system (53). In this system, complementation of a pair of split protein tags allows binding and fluorescence of an exogenously applied dye, which is nonfluorescent when unbound. Because splitFAST complementation is rapid and reversible, it is ideal for studying ERMCS stability. This is accomplished by tagging an ER targeting sequence with one component (NFAST) and an OMM targeting protein with the other (CFAST), allowing the measurement of the number and duration of ERMCS that are <25 nm. We transfected EFHD1-HA and *EFHD1^–/–^* HepG2 cells with ER-long-RspA-NFAST and OMM-short RspA-CFAST, incubated with the splitFAST fluorogen HMBR, and monitored fluorescence after ATP-induced ER Ca^2+^ release. We quantified the number of fluorescent spots and the duration of their fluorescence (Supplemental Figure 5, I–K). Remarkably, we found far fewer fluorescent spots formed in the *EFHD1^–/–^* cells compared with the EFHD1-HA cells, suggesting a failure to form ERMCS. When fluorescent spots did form, we found that their lifetime was shorter in *EFHD1^–/–^* cells compared with WT cells. Taken together, these results independently and directly confirm that EFHD1 helps stabilize ERMCS.

Finally, we directly measured the Ca^2+^- and INF2-dependent cellular actin response using a well-established protocol (41, 42). Here, ionomycin increases cytoplasmic Ca^2+^ to drive actin polymerization. With this assay, we visualized as a peri-nuclear actin ring in WT hepatocytes, but it was absent in *Efhd1*^–/–^ hepatocytes (Figure 3, H and I). We conclude from these experiments that EFHD1 is a spatiotemporal coincidence detector that helps create stable ERMCS. ER Ca^2+^ release is the trigger for EFHD1 to bundle actin from opposite membranes, efficiently tethering ER to mitochondria (Supplemental Figure 6). In the absence of EFHD1, ERMCS tethering requires a direct but unstable INF2-Spire1C interaction, which produces narrower ERMCS that are more prone to disruption.

### EFHD1 ablation reduces hepatocyte inflammation and the integrated stress response.

Next, we used a multiomic approach to define the link connecting remodeling to MASH. Here, we primarily used male mice, since differences in their liver phenotypes were strongest. We performed RNA-seq of total RNA of entire livers of WT and *Efhd1^–/–^* mice fed either normal or MASH diets (Supplemental Figure 7, A–D, and Supplemental Tables 1 and 2). MASH diet–fed animals had more pronounced differences between WT and *Efhd1^–/–^* compared with normal chow, but in both diets the most notable effect of EFHD1 deletion was a decrease in interferon inflammatory pathways (Figure 4, A and B). We also performed label-free proteomic analysis of entire livers (Supplemental Figure 7, E–G, and Supplemental Tables 3–5). We analyzed these data in 3 ways: looking at changes between WT and *Efhd1^–/–^* for each diet condition (Figure 4, C, E, F, and H) but also examining changes in WT between normal and MASH diets (Figure 4, D and G). This additional analysis allowed us to examine which specific pathways dysregulated by MASH are rescued by EFHD1 ablation (Figure 4, I–L). Using REACTOME pathway analysis, the most prominent changes caused by MASH versus normal diets were increased fibrosis, decreased mitochondrial metabolism, and complement pathway activation (Figure 4, D and G). For *Efhd1^–/–^* livers, we again found decreases in inflammatory and fibrosis pathways and improved mitochondrial metabolism.

To examine such changes further, we plotted the fold-change for each pathway in the “WT MASH versus WT normal” comparison against the fold-change in that pathway in the “*Efhd1^–/–^* MASH versus WT MASH” comparison (Figure 4, I–L). A negative slope shows that pathways upregulated by MASH in WT animals are downregulated in *Efhd1^–/–^* livers, and vice versa, confirming that pathways dysregulated in MASH tend to recover in the *Efhd1^–/–^* animals globally (Figure 4I). Focusing on specific parental REACTOME groups of interest, we found that fat metabolism was one cluster that did not show a significant relationship (Figure 4J). In striking contrast, broad increases in immune system pathways during MASH were downregulated in *Efhd1^–/–^* animals (Figure 4K), consistent with the histopathological phenotypes.

An unexpected and intriguing set of MASH-dysregulated pathways rescued by EFHD1 ablation were those related to translation (Figure 4, E, H, and L). Global decreases in mRNA translation are a hallmark of the integrated stress response (ISR). In the ISR, phosphorylation of the translation initiation factor 2α (EIF2α) globally downregulates translation, with preferential expression of ATF4-dependent genes that promote cellular recovery (54). Whereas temporary ISR activation is protective, prolonged activation promotes cell death. The ISR and a closely related mechanism, the unfolded protein response, are activated in the liver by ER lipotoxicity (55). The ISR can also be activated by severe mitochondrial dysfunction, dsRNA associated with viral infection, or starvation (54). In our proteomic dataset, translation was decreased in MASH, consistent with ISR activation, and rescued by ablating EFHD1, suggesting reductions in ISR in *Efhd1^–/–^* mice. Taken together, these data are consistent with histopathological and GWAS data suggesting that EFHD1 inhibition primarily prevents the hepatocyte injury leading to inflammation and fibrosis, with much less pronounced effects on lipid metabolism. Furthermore, these data point toward the ISR as a possible mechanism linking mitochondrial function to hepatocyte injury.

### Activation of protein kinase RNA-activated is via pathological release of mt-dsRNA.

To investigate the ISR further, we assessed the expression and phosphorylation state of EIF2α, upstream kinases, and ATF4 target genes (56). As expected, in MASH, we found substantial upregulation of EIF2α phosphorylation, PERK expression and phosphorylation, and ATF4 targets (Figure 5, A and B, and Supplemental Figure 8A). In contrast, compared with WT, *Efhd1^–/–^* livers on a MASH diet showed reduced ISR (Figure 5, C and D, and Supplemental Figure 8B).

Our next goal was to define the damage mechanism activating the ISR. Notably, a well-established inflammation trigger in other cell types, release of mitochondrial DNA (mtDNA), fails to activate an inflammatory response in hepatocytes because they lack STING expression (57–59). Another mitochondrial damage–associated signal may arise from improper processing of mtRNA. Normally, this RNA is transcribed as 2 separate but complementary strands, each cleaved to release specific transcripts. During mitochondrial dysfunction, mtRNA is inadequately processed and can hybridize and leak into the cytoplasm as a long dsRNA (60, 61). Within the cytoplasm, mt-dsRNA may be recognized by the dsRNA sensor PKR (protein kinase RNA-activated; *EIF2AK2* gene), which can trigger the ISR independently of PERK (54). Notably, hepatic PKR typically senses dsRNA from viruses such as hepatitis C, activating ISR to prevent viral replication (62). Thus, mt-dsRNA–dependent PKR activation may represent a maladaptive response.

The possibility that PKR might link mt-dsRNA to the ISR during MASH was interesting for additional reasons. Notably, we saw marked increases in PKR and p-PKR in MASH diet–fed mice, comparable to the increases in PERK (Figure 5, A and B, and Supplemental Figure 8A). Moreover, whereas PERK signaling was not affected by EFHD1 deletion, we saw decreases in p-PKR in MASH-fed *Efhd1^–/–^* mice relative to WT (Figure 5, C and D). In contrast, 2 other dsRNA sensors involved in innate immunity, RIGI and MDA5, were not substantially altered (Supplemental Figure 8, C and D). Finally, prior studies investigating overnutrition found that PKR deletion reduced organismal inflammation, but disagreed on whether lipid metabolism was affected, and failed to identify a trigger for PKR activation (63, 64). This pattern of protection from inflammation with variable effects on metabolism mimics the phenotype of EFHD1 ablation.

Therefore, to determine whether mt-dsRNA triggered the ISR in MASH, we stained human liver tissue sections for dsRNA (60). This revealed a clear increase in dsRNA signal during early MASH (Figure 5, E and F). In later stages, the dsRNA signal became more heterogenous but remained elevated. Widespread cytoplasmic dsRNA staining was also evident in isolated hepatocytes from WT mice fed a MASH diet (Figure 5G and Supplemental Figure 8, E and F) (65). In contrast, there was minimal dsRNA staining in both chow-fed WT mice and *Efhd1^–/–^* mice fed a MASH diet. The staining was specific for dsRNA, as it was absent when cells were treated with a dsRNA-cleaving endoribonuclease prior to antibody incubation (Supplemental Figure 8G). We then costained for both dsRNA and PKR in human and mouse livers (Figure 5, H and I, and Supplemental Figure 8, H and I). In healthy tissue, PKR localized to condensed nuclei in nonhepatocytes, likely leukocytes (66). Conversely, in MASH, PKR staining was now also present in hepatocytes and overlapped the cytoplasmic dsRNA stain. Therefore, in both humans and mice, overnutrition triggers the cytoplasmic release of endogenous dsRNAs, where they are sensed by PKR.

Deletion of EFHD1 prevented the pathologic increase in cytoplasmic dsRNA, suggesting mitochondrial origin. To confirm this, we immunoprecipitated either dsRNA or PKR from isolated hepatocytes and quantified bound mtRNA via quantitative reverse transcription polymerase chain reaction (61). Compared with nucleus-encoded genes (*Mcu* or *Gapdh*), mtRNAs were profoundly enriched in the dsRNA- or PKR-immunoprecipitated fraction in MASH-fed WT mice (Figure 5, J–M), including from cytoplasmic fractions (Supplemental Figure 8J). However, in *Efhd1^–/–^* mice fed a MASH diet, the degree of mt-dsRNA enrichment was much reduced (Figure 5, K and M). Finally, we found that dsRNA localized near DRP1 and mitochondria in MASH livers, linking mt-dsRNA release to excessive constriction (Figure 5N). In *Efhd1^–/–^* MASH hepatocytes, though dsRNA could be visualized when increasing image acquisition settings, the distance to DRP1 was increased (Figure 5O). Taken together, our data suggest that EFHD1 upregulation during MASH drives ERMCS hyperstability and mitochondrial fragmentation, causing the release of mt-dsRNA and the subsequent activation of a maladaptive PKR-dependent ISR.

### Mendelian randomization studies support a causal relationship between human PKR (EIF2AK2) and liver disease.

To investigate whether PKR produces a similar phenotype to EFHD1 in humans, we first queried the Common Metabolic Diseases Knowledge Portal (67). As with *EFHD1*, variation in the *PKR* (*EIF2AK2* gene) locus was associated with serum liver enzymes but not liver fat or other lipid metabolism parameters (Supplemental Figure 8K). Furthermore, analyzing the UK Biobank, we identified statistically significant associations (*P* < 8.87 × 10^–6^) between 35 genetic variants spanning a 105.8 kilobase region at the *EIF2AK2* locus and serum AST levels (Supplemental Figure 8L and Supplemental Table 6). Significant expression quantitative trait loci (*P* < 8.23 × 10^–4^) for *EIF2AK2* expression in liver were observed at 34 of these AST-associated variants (Supplemental Figure 8M) (68). Twenty-one of these variants were also predicted by RegulomeDB rank and model prediction scores to have a high likelihood of being functional (69).

To further support a causal association, Mendelian randomization (MR) was performed on these 35 variants at the *EIF2AK2* locus, using AST as the exposure and liver disease diagnosis as the outcome. MR is a causal inference method that uses genetic variants as instrumental variables to test the causal effect of that trait on a disease outcome. By leveraging the random assortment of alleles at conception, MR minimizes confounding and reverse causation, providing unbiased evidence for a causal link in humans. We ran 3 MR analyses that varied by liver diagnosis inclusion (Figure 5P and Supplemental Tables 7–11). Tier 1 was the broadest, including diagnoses associated with acute or chronic liver disease, malignancies, and infectious causes. In tier 2, we excluded infectious causes, including viral hepatitides. Tier 3 was the most restrictive, including only codes associated with chronic liver disease and excluding infectious causes. MR analysis for all 3 outcomes suggested a causal association between AST-associated variants and liver disease. Strikingly, the magnitude of the penalized inverse variance-weighted estimate for tier 3 was significantly larger than for tier 1, suggesting a stronger effect of PKR on chronic liver disease not due to viral infection. Overall, this putative causal relationship reinforces a pathway linking aberrant mt-dsRNA release to hepatocyte injury.

### Liver-specific EFHD1 inhibition in humans and mice is hepatoprotective.

Our final goal was to establish whether liver-specific EFHD1 inhibition could be hepatoprotective. As expected, in mice with liver-specific EFHD1 ablation (*Efhd1^hKO^*), hepatocytes had elongated mitochondria resistant to Ca^2+^-induced fission (Figure 6, A–D, and Supplemental Figure 9, A and B). Then, we injected 8- to 10-week-old male *Efhd1^hKO^* or *Alb*-Cre control mice with carbon tetrachloride (2 μL/g CCl_4_, 3 times/wk) intraperitoneally for 6 weeks, a chemical liver injury model that produces marked fibrosis rather than steatosis (70). Again, *Efhd1^hKO^* mice had reduced serum liver enzymes and fibrosis (Figure 6, E–I). This result revealed that EFHD1 mediates a core injury response independent from the initial insult.

Next, we investigated whether acute inhibition of EFHD1 could confer similar protection. First, we examined human 3D liver microtissues that were transduced before aggregation with either a nontargeting control or *EFHD1-*targeting short hairpin RNA (~85% knockdown of transcripts), packaged in adeno-associated virus (AAV-DJ, Supplemental Figure 9, C and D) (71). All viruses also encoded GFP, allowing us to confirm robust liver transduction. Both microtissue triglyceride content and TNF-α secretion were reduced, suggesting that acutely targeting EFHD1 could benefit inflammation and injury in these human models (Figure 6, J and K).

Second, 6-week-old male mice were fed a MASH diet for 18 weeks before a single injection of *Efhd1-*targeting short hairpin RNA, or a nontargeting control, packaged in a hepatotrophic AAV serotype (AAV8) (Supplemental Figure 9, E and F). Livers were assayed 12 weeks later. This protocol allowed us to observe the effects of *Efhd1* inhibition after MASH diet–induced injury (72). We used 2 independent shRNAs targeting *Efhd1*, with sh*Efhd1*#1 producing greater inhibition than sh*Efhd1*#2 (Supplemental Figure 9, G–J).

After *Efhd1* inhibition we again observed substantial decreases in liver enzymes, indicative of reduced liver injury (Figure 6, L and M). Unexpectedly, serum triglycerides as well as direct measures of lipid content, though not histopathological scores, also showed decreases, suggesting that acute EFHD1 inhibition led to downstream improvements in metabolism by preventing hepatocyte injury (Supplemental Figure 9, K–N). Finally, both histological and direct scoring showed improvements in inflammation and fibrosis (Figure 6, N–S, and Supplemental Figure 9O). In all these analyses, sh*Efhd1*#1 tended to produce stronger effects than sh*Efhd1*#2, consistent with a dose-response relationship between the degree of *Efhd1* inhibition and hepatic protection. Taken together, inhibiting EFHD1 is hepatoprotective across multiple human and mouse models of liver disease.

## Discussion

Interorganellar communication is essential for cellular homeostasis, but the fundamental principles governing the establishment of these contact sites remain poorly understood. Here, we identify EFHD1 as a factor that creates stable ERMCS by transducing a timing signal, ER Ca^2+^ release, into mechanical durability via actin bundling. A coincidence detection system integrating spatial proximity with a temporal cue provides several advantages over the current model of tethering driven by constitutive membrane–membrane interactions (Supplemental Figure 6). First, such interactions may be relatively inefficient, since these 2 proteins reside on different membranes. In support of this, ER and mitochondria in *Efhd1^–/–^* hepatocytes needed closer contact to establish a connection, evident in narrower ERMCS distances at baseline (Figure 3G). Second, by requiring Ca²^+^ binding, EFHD1 ensures that contact formation is event driven, occurring only at locations where the ER signals to mitochondria, and thereby preventing inappropriate or excessive contact during organelle motility in a crowded cytoplasm. Third, because EFHD1 transduces this signal specifically by actin bundling, it provides a way to generate mechanical strength at contact sites, evident in the disruption of ERMCS during metabolic stress in *Efhd1^–/–^* hepatocytes. Taken together, our findings reveal the molecular logic for robust ERMCS stabilization.

Multiple lines of evidence suggest EFHD1 does not directly affect lipid metabolism. In our data, EFHD1 inhibition did not substantially affect organismal lipid metabolism or energy balance. Though we found that acute EFHD1 inhibition improved serum triglycerides, and there was less steatosis in *Efhd1^–/–^* mice compared with controls on a normal diet, this difference disappeared in the hepatotoxic diets. Moreover, across different cell types, there is no consistent effect on metabolism after EFHD1 inhibition (12–15). Fatty acid synthesis genes were upregulated in liver organoids, but lipogenesis was unaffected in HepG2 cells (12, 13). Protein interaction compendia provide no support for direct EFHD1 binding to enzymes of lipid metabolism or glycolysis (Supplemental Figure 3A and Supplemental Figure 5D), and human *EFHD1* variants associate with markers of liver injury rather than hepatic fat content, closely mirroring the injury-predominant phenotype we observe across multiple mouse–diet models. Instead, EFHD1 directly functions as a Ca²^+^-dependent actin cross-linker, supported by protein interaction data, in vitro biochemistry, AlphaFold3 modeling, similarity to the EFHD2 homolog, and actin-binding and cross-linking assays. Thus, because EFHD1 inhibition during MASH produces variable lipid phenotypes, its effects on metabolism are indirect, occurring possibly via changes to mRNA translation due to the ISR or altering lipid transfer at ERMCS.

In contrast, EFHD1-dependent ERMCS stabilization creates an intrinsic vulnerability that directly leads to hepatocyte injury across all the models and conditions. EFHD1 upregulation during metabolic stress leads to pathological contact hyperstability, excessive mitochondrial fragmentation, and mt-dsRNA leakage. In the liver, cytoplasmic dsRNA is typically encountered during viral infection, where PKR drives the ISR to prevent translation of viral RNA. Here, we establish that during MASH, pathological mt-dsRNA release causes maladaptive activation of this pathway (Supplemental Figure 9P). We establish a candidate causal relationship between PKR and liver injury in humans, a relationship that is surprisingly strongest for noninfectious chronic liver disease. Inhibiting EFHD1 protects the liver by selectively dampening this PKR-dependent ISR without disrupting PERK-driven responses. Because EFHD1 is a central node integrating Ca^2+^ signaling, organelle dynamics, and immune activation, targeting its activity provides a therapeutic strategy fundamentally different from metabolic modulation for the treatment of liver injury.

## Methods

Detailed methods are available in the Supplemental Methods.

### Sex as a biological variable.

Analyses of liver function in mice are shown for both sexes, and similar findings are reported for both sexes. Because female mice are less injured by overnutrition (26), we performed proteomic analyses and liver-specific analyses of EFHD1 inhibition on male mice only. This allowed us to efficiently gain insight into the affected pathways while minimizing the use of animals.

### Statistics.

Microsoft Excel, OriginPro (OriginLab), and R Studio (Posit Software) were used for data analysis. Figures were assembled in Adobe Illustrator v30. For 2-sample comparisons where *n* < 15 or where a Shapiro-Wilk normality test found a non-normal distribution, we used a Mann-Whitney *U* nonparametric test. For comparisons on samples with normally distributed data sets or where *n* > 50, we used a 2-tailed, unequal-variance, Student’s *t* test (73). For comparisons of multiple samples, a 1-way ANOVA was performed for normal data and a Kruskal-Wallis ANOVA test for non-normal data. For analysis of fluorescence datasets consisting of repeated measures from individual mice, we utilized a linear mixed effects model in R to account for the hierarchical nature of the data, as described (74). *P* values of less than 0.05 were considered significant.

### Study approval.

The study protocol for human liver tissue sections was reviewed by the Institutional Review Board at HCI and determined to be exempt from human subjects research (exemption number IRB_00091019). All animal procedures have been reviewed and approved by the Institutional Animal Care and Use Committee at the University of Utah, and for the whole-mitoplast electrophysiology experiments, at the University of Maryland Baltimore.

### Data availability.

RNA-seq data have been deposited at the NCBI Gene Expression Omnibus database under record GSE301924. The mass spectrometry proteomics data have been deposited to the ProteomeXchange Consortium via the PRIDE partner repository with the dataset identifier PXD069152. All raw data values are included in the Supporting Data Values file.

## Author contributions

DRE, ECR, and DC conceived the study. DRE, ECR, YBM, DS, TRP, EB, CJS, RB, SM, NH, DS, FV, JZ, PNM, SF, VG, WLH, MGP, KJE, and DC developed methodology. DRE, ECR, YBM, HED, DM, AMV, DS, TRP, SHJL, EB, NKR, ARB, AMB, CJS, RB, SM, NH, XY, AC, AK, DMN, DS, VDN, VV, KHC, FV, JZ, PNM, SB, KJE, and DC investigated. DRE, DS, TRP, VG, MGP, and DC performed visualization. DRE, AK, DMN, PNM, ASA, RMS, SAS, and DC acquired funding. DRE, KJE, and DC performed project administration. DRE, PNM, SB, SGD, ASA, SF, VG, RMS, WLH, SAS, MGP, JR, KJE, and DC supervised. DRE and DC wrote the original draft. DRE, DS, TRP, EB, CJS, RB, JZ, PNM, SB, SGD, ASA, VG, RMS, WLH, SAS, MGP, JR, KJE, and DC reviewed and edited the manuscript.

## Conflict of interest

DRE and DC are inventors on provisional patent PCT/US2025/055547 filed by the University of Utah that covers the pathways discussed here. SAS is cofounder and shareholder of Centaurus Therapeutics. JR is a founder of Vettore Biosciences and a member of its scientific advisory board.

## Funding support

This work is the result of NIH funding, in whole or in part, and is subject to the NIH Public Access Policy. Through acceptance of this federal funding, the NIH has been given a right to make the work publicly available in PubMed Central.

- National Institutes of Health grants R01HL165797, R01HL141353, R01DK141142 to DC.
- Larry H. Miller Driving Out Diabetes Initiative grant to DC.
- Nora Eccles Treadwell Foundation grant to DC.
- National Institutes of Health grants R01HL174450, R01HL177965 to ASA.
- National Institutes of Health grant R01GM145806 to VG.
- National Institutes of Health grant R01DK128819 to PMN.
- National Institutes of Health grant T32AR007592 to DMN.
- National Institutes of Health grant R01HL152691 to RMS.
- National Institutes of Health grants U01CA272529, R01DK131609, R01DK115824, R01HL170575, R01DK130296 to SAS.
- National Institutes of Health grant T32DK091317 to TRP.
- American Diabetes Association grant 11-22-ICTSPM-09 to MGP.
- American Heart Association Postdoctoral award 834544 to DRE.
- American Heart Association/Barth Syndrome Foundation Postdoctoral award 24POST1241582 to AK.

## Supplementary Material

Supplemental data

Supplemental tables 1-11

Supporting data values

## Figures and Tables

**Figure 1:**
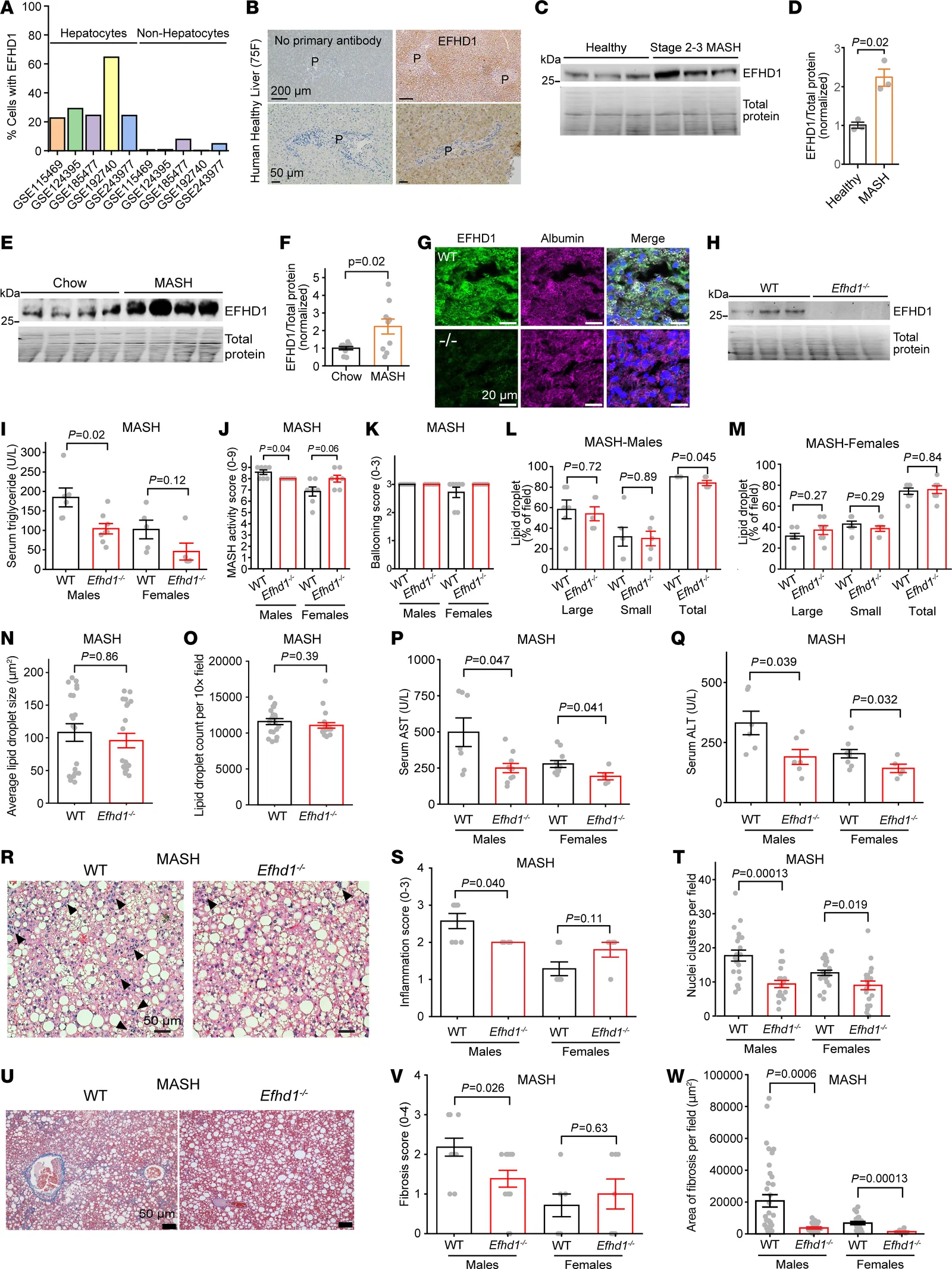
Loss of EFHD1 prevents hepatocyte injury in MASH. (**A**) Summary of human liver single-cell RNA-seq studies. (**B**) Immunohistochemistry showing EFHD1 is enriched around portal tracts (P) in healthy human liver. 75F, 75-year-old female. (**C** and **D**) Western blot (**C**) and summary (**D**), human MASH. (**E** and **F**) Western blot and summary, MASH diet mice. (**G**) EFHD1 (green) coexpression with the hepatocyte marker, albumin (magenta), in WT or *Efhd1^–/–^* livers. In merge, nuclei are DAPI-stained. (**H**) EFHD1 is absent in *Efhd1^–/–^* livers. (**I**–**W**) Assays performed on mice following 28 weeks of MASH diet. (**I**) Serum triglyceride levels. (**J**) MASH activity score. (**K**) Hepatocyte ballooning score. (**L** and **M**) Histopathological assessment of liver fat. (**N** and **O**) Direct measurement of lipid droplet size (**N**) and count (**O**). (**P** and **Q**) Serum aspartate aminotransferase (AST) and alanine aminotransferase (ALT). (**R**) Liver hematoxylin and eosin staining. Arrows, leukocyte clusters. (**S**) Histopathological inflammation score. (**T**) Liver leukocyte nuclei cluster counts. (**U**) Liver Masson’s trichrome staining. Blue, fibrosis. (**V**) Histopathological fibrosis score. (**W**) Fibrotic area from liver micrographs. All clinical histopathological scoring in **J**–**M**, **S**, and **V** was assessed in blinded fashion by a liver pathologist. For **N** and **O**, both sexes pooled. *t* test: **D**, **F**, **I**, **L**, **M**, **P**, **Q**, and **T**. Mann-Whitney *U* test: **J**, **L**, **M**–**P**, **S**, **V**, and **W**. *N* values, figure panel in parentheses. Human liver: 6(**D**). Chow: 10(**F**); MASH: 10(**F**). WT-M: 6(**I**, **L**, and **Q**), 7(**J**, **K**, **P**, and **S**), 21(**T**), 11(**V**), 36(**W**); *Efhd1^–/–^*-M: 8(**I**), 6(**J**, **K**, **Q**, and **S**), 5(**L**), 10(**P**), 20(**T**), 13(**V**), 21(**W**); WT-F: 5(**I**), 7(**J**, **K**, **M**, **S**, and **V**), 11(**P**), 9(**Q**), 24(**T**), 21(**W**); *Efhd1^–/–^*-F: 5(**I**, **P**, **Q**, and **S**), 7(**J**, **K**, **M**, and **V**), 21(**T**), 12(**W**). WT: 21(**N** and **O**); *Efhd1^–/–^*: 21(**N** and **O**). Bars: mean ± SEM.

**Figure 2:**
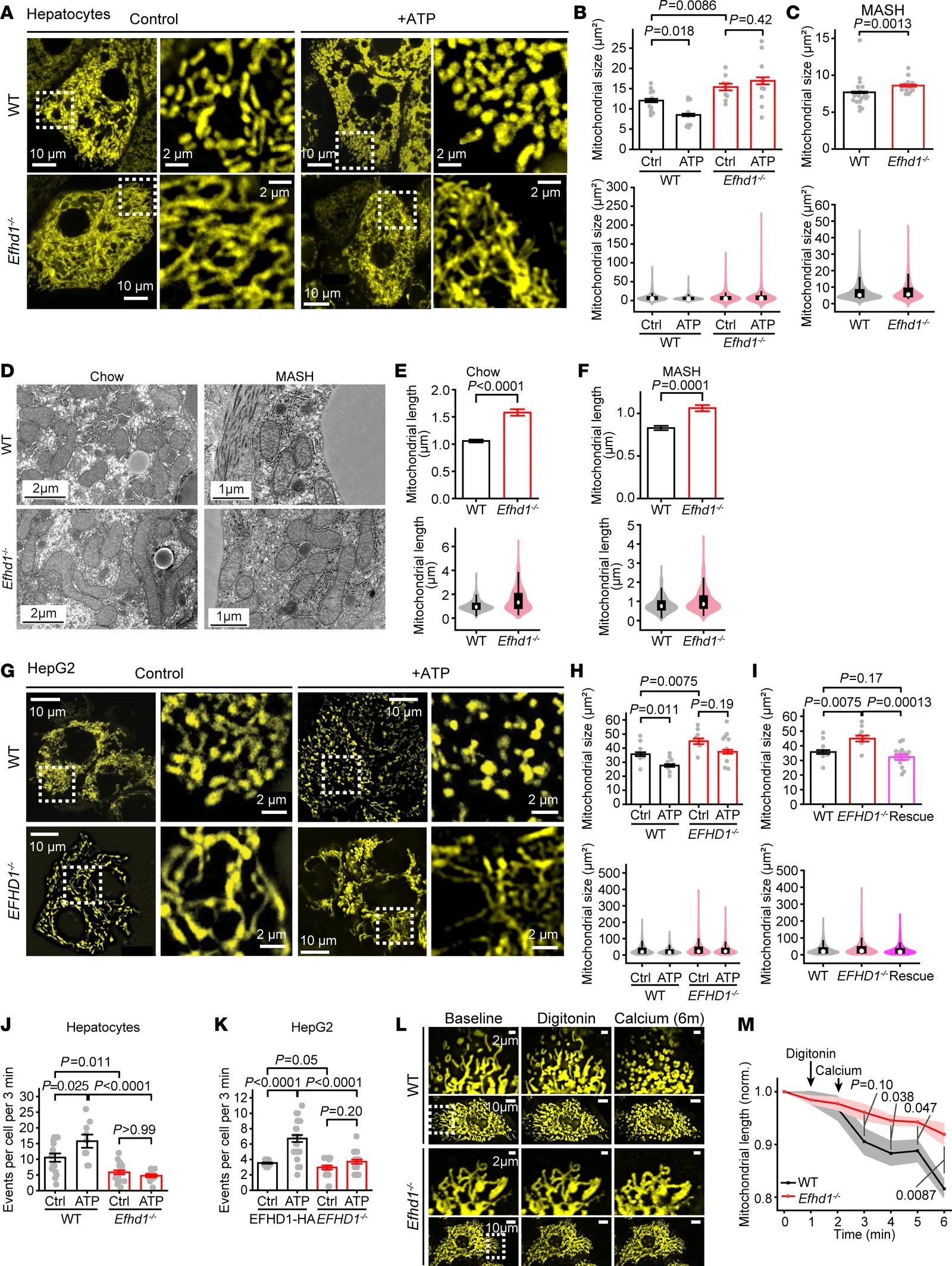
EFHD1 is necessary for Ca^2+^-induced mitochondrial remodeling. (**A**) Representative hepatocytes, 200 nM MitoTracker Orange staining before (control) or 5 minutes after treatment with 1 mM ATP (+ATP). Right, boxed insets at higher magnification. (**B**) Automated iLastik analysis, hepatocyte mitochondrial size. Data are displayed as a bar chart (top) to show mean effects and as a violin plot (bottom) to show the full distribution. Dots in bar chart represent the average value within each cell. (**C**) Hepatocyte mitochondrial size after 28 weeks of MASH diet, with data displayed as in **B**. (**D**) Representative TEM of mouse livers. (**E** and **F**) Hepatocyte mitochondrial length from TEM. (**G**) As in **A**, but for WT or *EFHD1^–/–^* HepG2 cells. (**H**) As in **B**, but for HepG2 cells. (**I**) Mean mitochondrial size. WT and *EFHD1^–/–^* are same as in **H**, ctrl condition. (**J** and **K**) Fission events counted after vehicle (PBS, ctrl) or 1 mM ATP. Hepatocytes: MitoTracker label; HepG2: mito-mGold2s label. (**L** and **M**) Representative images (**L**) and summary of mitochondrial length (**M**). Top, boxed insets in **L** at higher magnification. Linear mixed effects model: **B**, **C**, **H**, and **I**, *t* test: **E**, **F**, and **M**. One-way ANOVA: **J** and **K**. *N* values, figure panel in parentheses. WT: 3,755 mitochondria(m) from 12 cells(c) from 4 animals(a) (**B**), 1226m 36 c(**H**); WT+ATP: 2,221m 9c 4a(**B**), 1,301m 27c(**H**); EFHD1-KO: 2,306m 10c 6a(**B**), 927m 25c(**H**); EFHD1-KO+ATP: 2,286m 10c 5a(**B**), 1,068m 27c(**H**). WT: 7,071m 19c 3a(**C**), 347(**E**), 274(**F**); EFHD1-KO: 4,642m 13c 3a(**C**), 273(**E**), 294(**F**). WT: 1,226m 36c(**I**); *EFHD1^–/–^* KO: 927m 25c(**I**); rescue: 530m 41c(**I**). WT: 15c(**J**); WT+ATP: 9(**J**); *Efhd1^–/–^*: 19(**J**); *Efhd1^–/–^*+ATP: 12(**J**). EFHD1-HA: 22 cells(**K**); EFHD1-HA+ATP: 29(**K**); *EFHD1^–/–^*: 19(**K**); *EFHD1^–/–^*+ATP: 21(**K**). WT: 14(**M**); *Efhd1*^–/–^: 9(**M**). Bars: mean ± SEM.

**Figure 3:**
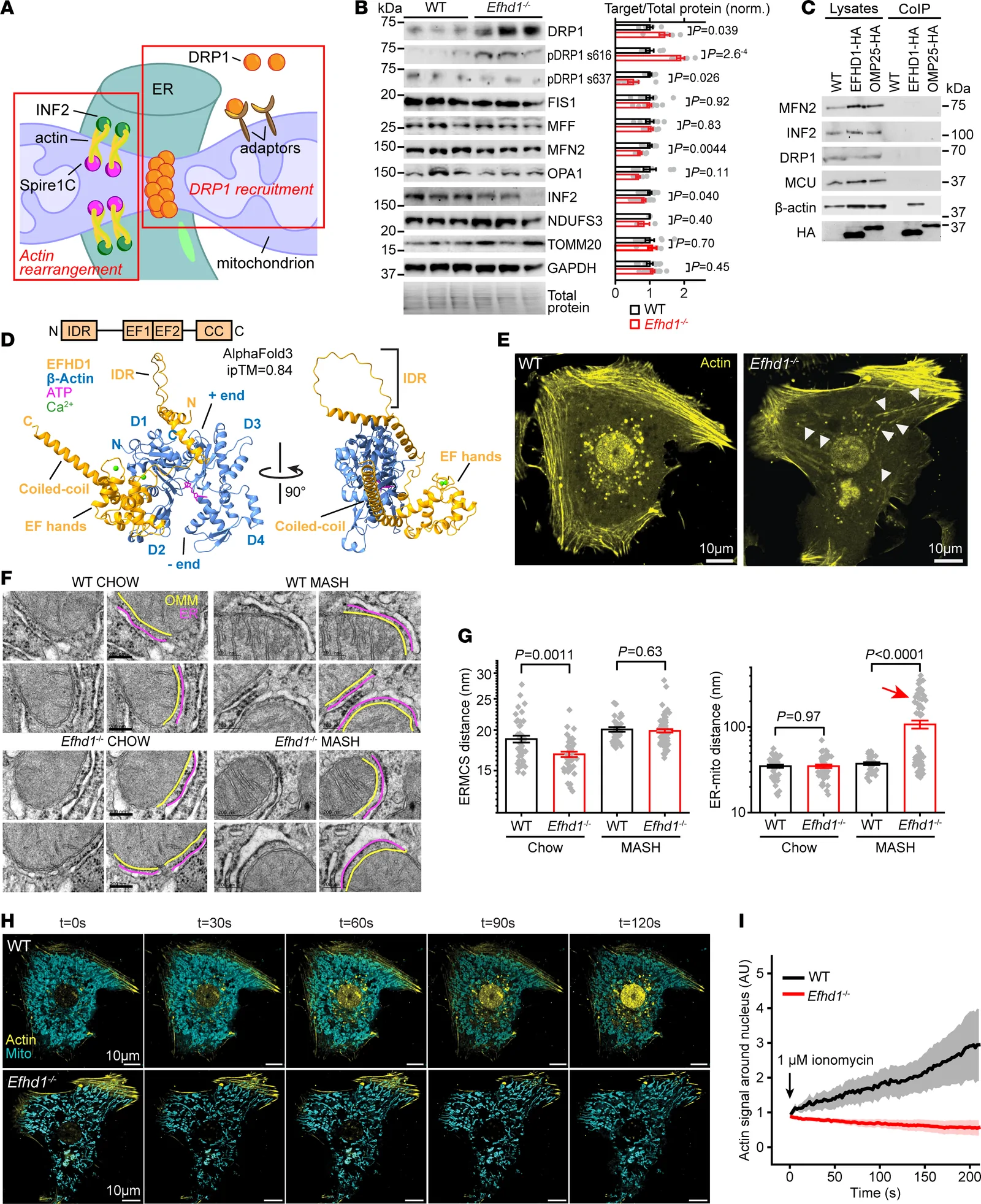
EFHD1 triggers Ca^2+^-dependent ERMCS stabilization. (**A**) Important events to establish ERMCS (actin rearrangements) and initiate fission (DRP1 recruitment). (**B**) Liver protein Western blot and summary. (**C**) Co-immunoprecipitation in HepG2 cells, untransfected (WT) or transfected with EFHD1-HA or OMP25-GFP-HA (outer membrane protein). Representative of 4 experiments. (**D**) High-confidence EFHD1:β-actin AlphaFold3 interaction prediction. ipTM, interface predicted template modeling. EFHD1 domains are shown above. ATP and Ca^2+^ are placed correctly within the EFHD1 EF hands and actin nucleotide binding pocket, respectively. (**E**) Representative SiR-actin–stained hepatocytes. Arrows, cytoplasmic stress fibers. (**F**) Representative TEM for ERMCS. Left, plain images. Right, OMM (yellow) and ER membrane (magenta) at ERMCS highlighted. (**G**) ER-mitochondrial distance at ERMCS (left) or any close association (<500 nm) on TEMs. Arrow points to a new population in MASH *Efhd1^–/–^* hepatocytes. (**H** and **I**) Representative images (**H**, same cells as in Figure 2E) and summary (**I**), perinuclear SiR-actin signal in chow-fed hepatocytes. *t* test: **B** and **G**. *N* values, figure panel in parentheses. WT: 3–11(**B**); *Efhd1^–/–^*: 3–14(**B**). ERMCS - WT chow: 43(**G**); *Efhd1^–/–^* chow: 45(**G**); WT MASH: 35(**G**); *Efhd1^–/–^* MASH: 81(**G**); ER-mito distance - WT chow: 42 micrographs(**G**); *Efhd1^–/–^* chow: 41(**G**); WT MASH: 33(**G**); *Efhd1^–/–^* MASH: 74(**G**). 3 mice per condition(**G**). WT: 3 mice, 11 cells (**I**). *Efhd1^–/–^*: 3 mice, 8 cells (**I**). Bars: mean ± SEM.

**Figure 4:**
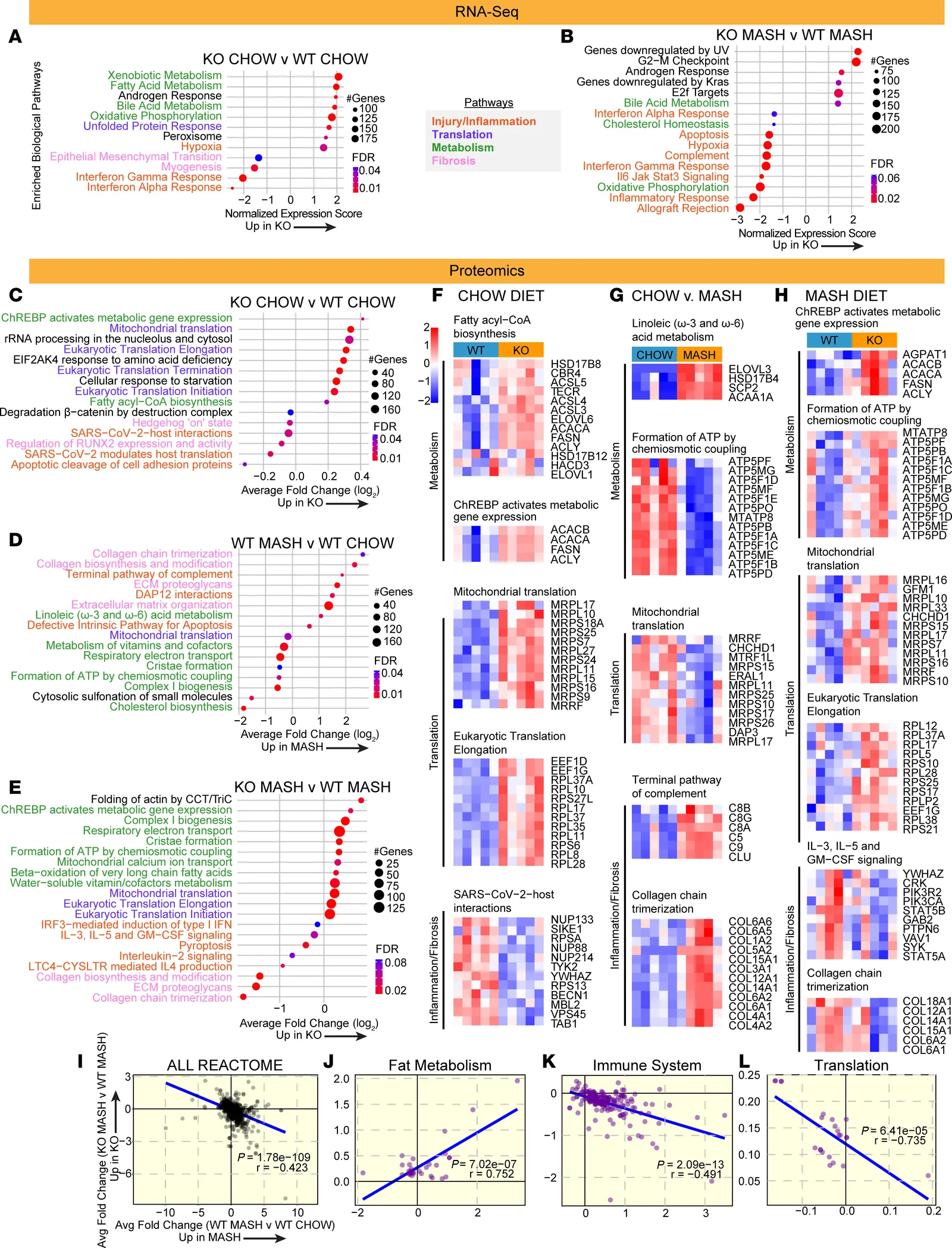
Transcriptomic and proteomic analysis of EFHD1 ablation. (**A** and **B**) Selected Hallmark pathways differentially regulated in whole-liver RNA-seq of *Efhd1^–/–^* versus WT mice. FDR, false discovery rate. (**C**–**E**) Selected REACTOME pathways differentially regulated in whole-liver proteomics. (**F**–**H**) Heatmaps of selected proteins from each indicated REACTOME pathway. (**I**–**L**) Fold-change for WT MASH versus WT normal chow (*x* axis) graphed against the fold-change for *Efhd1^–/–^* MASH versus WT MASH (*y* axis) for parental REACTOME pathways (**I**), fat metabolism pathways (**J**), immune system pathways (**K**), and translation pathways (**L**). Changes in MASH (relative to normal chow) reversed in *Efhd1^–/–^* are visible as a negative slope. For **K**, increases in immune pathways during WT MASH are decreased in *Efhd1^–/–^* MASH, whereas for **L**, decreases in translation during WT MASH are increased in *Efhd1^–/–^* MASH. Pearson’s correlation: **I**–**L**. RNA-seq, chow: WT: 5, *Efhd1^–/–^*: 5. MASH: WT: 4, *Efhd1^–/–^*: 5. Proteomics, chow: WT: 5, *Efhd1^–/–^*: 5. MASH: WT: 5, *Efhd1^–/–^*: 5.

**Figure 5:**
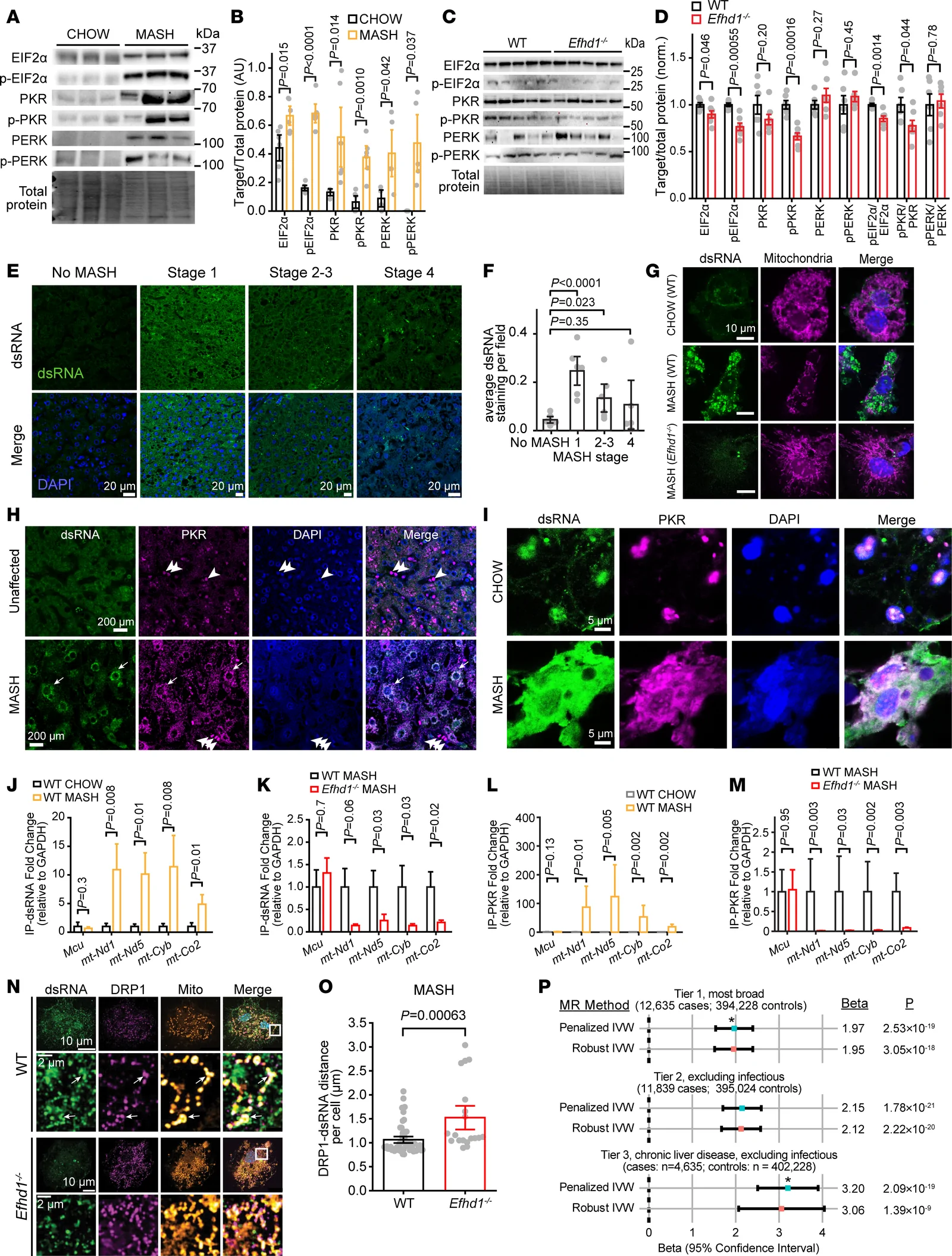
Excessive ERMCS stabilization triggers pathological mt-dsRNA release and a PKR-dependent ISR in MASH. (**A** and **B**) Western blot (**A**) and summary (**B**). EIF2α, eukaryotic translation initiation factor 2-α; p-EIF2α, phosphorylated EIF2α; PKR, protein kinase RNA-activated; p-PKR, phosphorylated PKR; PERK, PKR-like endoplasmic reticulum kinase/eukaryotic translation initiation factor 2-alpha kinase 3 (EIF2AK3); p-PERK, phosphorylated PERK. (**C** and **D**) MASH diet Western blot (**C**) and band quantification (**D**). (**E** and **F**) Representative images (**E**) and quantification summary (**F**) of dsRNA immunohistochemistry in human liver. (**G**) Isolated mouse hepatocyte immunohistochemistry. (**H**) Representative high-contrast immunohistochemistry of human livers. Arrowheads, PKR in dense nonhepatocyte nuclei; arrows, dsRNA- and PKR-stained cytoplasmic signal. (**I**) Representative images, isolated mouse hepatocytes. (**J**–**M**) Quantitative RT-PCR following immunoprecipitation of dsRNA (**J** and **K**) or PKR (**L** and **M**) from isolated mouse hepatocytes. (**N**) Representative immunocytochemistry of isolated hepatocytes from MASH diet mice. Because less dsRNA was evident in *Efhd1^–/–^* hepatocytes, image acquisition settings for these were adjusted to maximize dsRNA signal. Bottom, inset at higher magnification. (**O**) Average dsRNA-DRP1 distance per hepatocyte. (**P**) Penalized and robust inverse variance-weighted MR analyses support a causal relationship between human *PKR* (*EIF2AK2*) and liver disease, using AST as the exposure. *, 2 distributions with non-overlapping 95% confidence intervals. *t* test: **B** and **D**. Mann-Whitney *U* test: **B**, **J**–**M**, and **O**. Linear mixed-effect model: **F**. *N* values, figure panel in parentheses. Chow: 3–6(**B**); MASH: 5–6(**B**). WT: 6(**D**); *Efhd1^–/–^*: 6–7(**D**). Stage 0, 1, 2–3: 6(**F**); Stage 4: 5(**F**). Chow: 8(**J**), 5(**L**); MASH: 6(**J**), 5(**L**). WT: 6(**K**), 5(**M**); *Efhd1^–/–^*: 8(**K**), 7(**M**). WT: 49(**O**); *Efhd1^–/–^*: 19(**O**). Bar: mean ± SEM.

**Figure 6:**
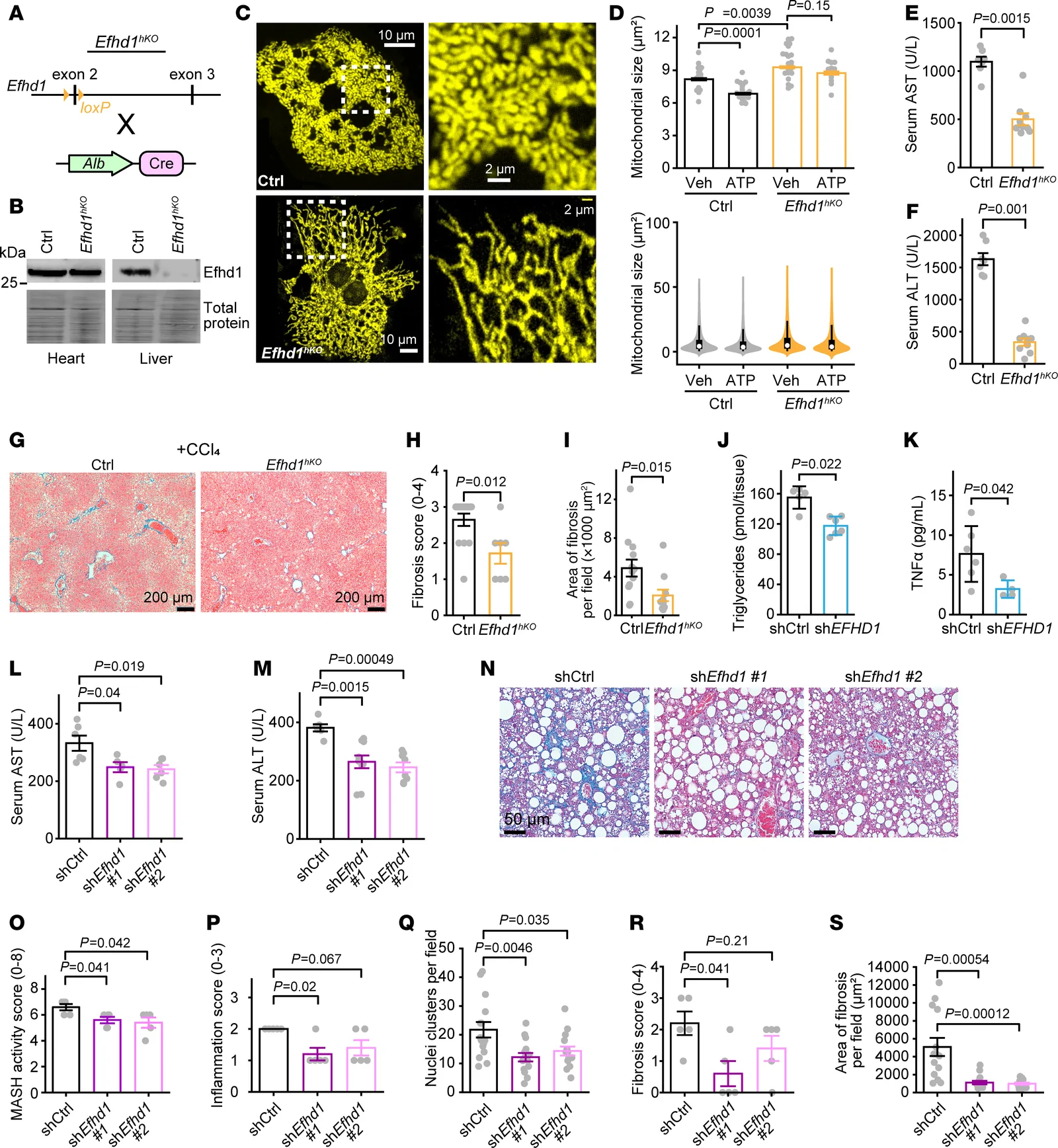
Liver-specific EFHD1 inhibition reduces hepatocyte injury. (**A**) Hepatocyte-specific *Efhd1* deletion via frameshift and early termination. (**B**) Western blot where heart is a control. (**C**) MitoTracker-stained isolated mouse hepatocytes. Right, boxed insets at higher magnification. (**D**) Automated iLastik analysis. Bar chart (top) shows mean effects; violin plot (bottom) shows full distribution. Dots in bar chart, average value per cell. (**E**–**I**) Assays following 4 weeks of CCl_4_ injection. (**E** and **F**) Serum liver enzymes. (**G**) Liver Masson’s trichrome staining. Fibrosis, blue. (**H**) Histopathological fibrosis score. (**I**) Fibrotic area from liver micrographs. (**J** and **K**) Human microtissue (**J**) triglyceride and (**K**) TNF-α production. (**L**–**S**) Assays on MASH diet and AAV8 treatment. (**L** and **M**) Serum liver enzymes. (**N**) Liver micrographs of Masson’s trichrome staining. (**O**) MASH activity score. (**P**) Histopathological inflammation score. (**Q**) Liver leukocyte nuclei cluster counts. (**R**) Histopathological fibrosis score. (**S**) Fibrotic area measured from liver micrographs. **B**–**I** and **L**–**S** used male mice. Clinical histopathological scoring in **H**, **O**, **P**, and **R** was assessed in blinded fashion by a liver pathologist. Linear mixed effects model: **D**. Mann-Whitney *U* test: **E**, **F**, **H**–**K**, **O**, **P**, and **R**. One-way ANOVA: **L**, **M**, **Q**, and **S**. *N* values, figure panel in parentheses. WT Veh: 12,055 mitochondria(m) from 24 cells(c) from 6 animals(a) (**D**); *Efhd1^hKO^* Veh: 17,876m 22c 3a(**D**); WT ATP: 19,498m 20c 4a(**D**); *Efhd1^hKO^* ATP: 10,734m 18c 3a(**D**). WT: 7(**E** and **F**), 14(**H**), 13(**I**); *Efhd1^hKO^*: 9(**E** and **F**), 7(**H**), 11(**I**). shCtrl-human: 5(**J**), 6(**K**); sh*EFHD1*-human: 6(**J**), 4(**K**). shCtrl: 6(**L** and **M**), 5(**O**, **P**, and **R**), 15(**Q**), 14(**S**); sh*Efhd1-1*: 5(**L**, **O**, **P**, and **R**), 10(**M**), 15(**Q** and **S**); sh*Efhd1-2*: 6(**L**), 8(**M**), 5(**O**, **P**, and **R**), 15(**Q** and **S**). Bar: mean ± SEM.

## Version 1 09/01/2026

In-Press Preview

## Version 2 10/01/2026

Electronic publication
